# End-to-End Segmentation and Classification of Zooplankton Using Shadowgraphy and Convolutional Neural Networks

**DOI:** 10.3390/s26061824

**Published:** 2026-03-13

**Authors:** Andrew Capalbo, Francis Letendre, Alexander Langner, Abigail Blackburn, Owen Dillahay, Michael Twardowski

**Affiliations:** Harbor Branch Oceanographic Institute, Florida Atlantic University, 5600 US-1N, Fort Pierce, FL 34946, USA

**Keywords:** in situ imagery, zooplankton, shadowgraphy, segmentation, classification, machine learning, biodiversity

## Abstract

**Highlights:**

**What are the main findings?**

- Four CNN-based classification algorithms were successfully implemented for categorizing zooplankton with >95% accuracy using over 70k images.
- Niched networks are a tractable, modular and efficient avenue for specific taxonomic or functional classification.

**What are the implications of the main findings?**

- This segmentation and classification algorithm can potentially be used to study zooplankton interactions and various functional traits.
- Its high accuracy when given out-of-distribution data shows that the algorithm can be used in diverse ecosystems.

**Abstract:**

With in situ imaging systems becoming more common, precise, and economically viable, use of these systems has grown dramatically, including both automated classification and biomass estimations. However, a rather large and overlooked portion of these efforts is reliable detection and classification of these organisms as they pass through the imaging device. This paper focuses on the development of an end-to-end classification CNN-based algorithm for marine zooplankton using the in situ Ichthyoplankton Imaging System (ISIIS-DPI) from Bellamare (La Jolla, CA, USA). Our novel approach considers many issues with automated segmentation and classification, including over-segmentation, noise segmentation, and organism size input. This allows for classifications in diverse water types, demonstrated by the comparison of three datasets created in conjunction with this project, each with very different water properties and zooplankton communities (Florida Gulf coast; Trondheimsfjord, Norway; Sargasso Sea). Our segmented image dataset contains 70,624 regions of interest (ROIs) across four organism classes—Chaetognath, Crustacean, Gelatinous, and Larvacean—with two classes dedicated to detritus. Four common network architectures—Resnet, Xception, GoogleNet, and Darknet—are trained on this dataset, with final test accuracies in the range of 95.94–96.09%. Following this initial training, a secondary level of classification is introduced. The base Gelatinous class is further divided into six groups. The same four CNN architectures are used once again, with final accuracies in the range of 86.12–90.40%, showing the ability to taxonomically classify down to the order level. The present work introduces a versatile, adaptable, scalable and autonomous segmentation and classification algorithm using niched networks mirroring taxonomy, and is fully contained in a publicly available MATLAB R2025a custom graphical user interface.

## 1. Introduction

Zooplankton are a vital part of ocean ecosystems, acting as important diel vertical migrators and as a major link in food webs and the carbon pump [1]. Traditional sampling of this functional group usually consists of nets, pumps, and bottles, followed by time-consuming sample preservation, identification, and counting. However, this can result in large collection biases due to mesh size used, avoidance of sampling gear by larger zooplankton, and difficulty in collecting fragile organisms, i.e., gelatinous plankton [2,3]. In situ zooplankton imaging technologies have become much more prevalent in the field of oceanography, being advantageous for studying species distribution and behavioral ecology directly in the water column. Most systems make use of high-speed imaging cameras, with some of the most common instruments including the ZooScan (Hydroptic, L’Isle-Jourdain, France), Zoocam (IFREMER, Plouzané, France) [4], Underwater Vision Profiler (UVP, Hydroptic, L’Isle-Jourdain, France) [5], the AUTO-HOLO (HBOI, Fort Pierce, FL, USA) [6] and the ISIIS-DPI (In Situ Ichthyoplankton Imaging System Deep-Focus Particle Imager) (Bellamare, La Jolla, CA, USA) [7]. These systems can be moored, towed or profiled by a research vessel, and integrated into autonomous platforms such as Argo floats, remotely operated vehicles (ROVs) and autonomous underwater vehicles (AUVs) [8]. However, since a single research expedition will usually yield hours and terabytes of video data, getting a trained professional to go through these videos and manually segment and identify every planktonic species of interest is extremely inefficient, both in terms of cost and time. This work was motivated by the need for a tool to semi-automatically segment these organisms from video frames.

Recent advances in both imaging technologies and machine learning have yielded novel methods in organism identification and classification [9]. Drago et al. [10] and Panaïotis et al. [11] used in situ imagery databases coupled with a web-based taxonomic classification algorithm to estimate global zooplankton biomass and community composition. Additionally, considering that net sampling may often cause damage to delicate structures, such as swimming or filtering appendages, feeding tentacles, egg sacs, and mucus housings, which are often required for precise identification, sampling organisms using in situ imaging methods can help circumvent this bias. Unique morphological characteristics [8] and behaviors, such as predation, parasitism, and competition [12,13], can then be observed naturally in the water column. These systems can also provide insight into oceanic carbon flux by imaging marine snow aggregations and measuring sinking rates [14]. Johnsen et al. [15] showed that using in situ polarizing imagery can enhance contrast and highlight morphological traits in gelatinous organisms. In situ digital holography has been used to establish a preferential orientation bias in thin shear layers by phytoplankton and ciliates, possibly to increase photosynthesis efficiency [16,17,18]. Imaging the water volume also allows planktonic patchiness assessments [19] and determining nearest neighbor distances (NNDs) [20,21,22].

Attempting to identify different types of plankton from video data has proved exceedingly difficult, especially down to the family and genus levels, requiring one to distinguish between very specific patterns. Sufficient taxonomic details for precise classifications are not always resolved. Video frames are usually passed through various thresholding methods to identify regions of interest (ROIs), which are then categorized [23]. Machine learning has emerged as an effective methodological approach for exploiting available morphological information [9,24,25,26]. Unlike a typical computer program, in which the algorithm is written manually, a neural network can produce a functional model on its own by training itself on large datasets. In the case of image classification, convolutional neural networks (CNNs) are fed hundreds to thousands of labeled images and are then instructed to learn the distinguishing features on their own. A CNN does this by passing images through multiple functions using multiple sets of weights or parameters, eventually producing confidence ratios for each image. The CNN then looks at the correct categories for each image and calculates the loss, or how poorly it did. Using backpropagation, it can estimate how much each weight needs to be adjusted to increase its overall accuracy. Network training is done over batches of 128 images. An epoch is completed when the network has done a complete pass over the entire training dataset. This process is repeated either until a predetermined maximum number of epochs has been reached or until it stops improving; the trained model can then be exported and used.

Recent works have used these CNNs for a variety of tasks, including image classification and zooplankton biomass estimates. Bi et al. utilized Histogram of Oriented Gradients (HOG) features to build a unique zooplankton classification dataset [23]. Using images taken by the ZOOVIS system in Chesapeake Bay, ROIs were segmented from the initial frame using binary thresholding. Following this, a HOG algorithm was applied, generating a feature set of binned gradient data. They found that a standard vector machine (SVM) could classify with >80% precision; however, they continuously ran into problems with noisy image fields that interfered with classification. Bell and Hopcroft [27] compared several machine learning methods, including traditional CNNs, random forest classifiers, and K nearest neighbors. After testing each classifier, random forest was chosen due to its low k-fold cross validation on the training set. They found that this provided adequate classification, with particles reaching 81% accuracy and medium copepods (e.g., *Pseudocalanus* sp., *Acartia* sp.) reaching 73%. Kyathanahally et al. [28] utilized ensemble approaches, where multiple network predictions are used to make a final classification. They found an extremely high accuracy, ranging from 96% to 97% across their training sets. Rawat et al. [29] used a neural network (Inception v3) to extract a feature set from zooplankton images. This method relies on the inner layers of the network rather than classifying images; only the first portion of convolution and transformation layers is applied to the image. The output is a seemingly random dataset, but contains features representative of the original image. These data were then used as inputs for a set of classifiers which included traditional CNNs, standard vector machines (SVMs), and logistic regression. They found excellent results, with their model producing 99.5% accuracy on the training set. Machine learning is a powerful tool, and offers many avenues for its application. These works, while varying in terms of classifiers and datasets, all contribute to understanding plankton ecology, e.g., abundance and diversity, relationships, grazing patterns, and other insightful characteristics. As these organisms make up one of the largest communities on the planet, developing novel methods and sensors to study and monitor this functional group is a critical need.

**Figure 1:**
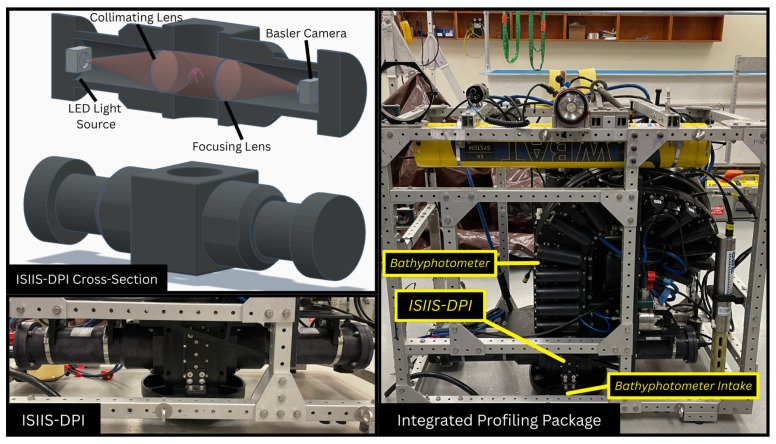
ISIIS-DPI Imager (bottom left) accompanied by a simplified model showing internal components (top left). The full instrumentation package is shown on the right panel, with the ISIIS-DPI imager located at the bottom, upstream of the bathyphotometer’s intake. Images are not to scale.

Data used to train our semi-autonomous classification algorithm were obtained using an Ichthyoplankton Imaging System Deep-Focus Particle Imager camera (ISIIS-DPI, Bellamare, CA, USA) deployed during three recent Harbor Branch Oceanographic Institute (HBOI) research expeditions in the Gulf of Mexico (March 2024), Trondheimsfjord, Norway (August 2024), and the Sargasso Sea (April 2025). These deployments were part of a larger project studying mechanically stimulated planktonic bioluminescence using a custom bathyphotometer developed at HBOI, where the ISIIS-DPI was integrated near the intake (Figure 1). The bathyphotometer consists of a 5″ inner diameter coiled flow-through tube with two linear arrays of photomultiplier tubes (PMTs) on opposing sides to measure bioluminescence emission kinetics. The high flow rate of the pump, i.e., 18 L/s, minimizes sensor avoidance by zooplankton and limits sampling biases of the water column community. The three field deployments had very distinct planktonic communities and optical water parameters, resulting in a taxonomically and optically diverse dataset (see Section 2.4.1 for detailed dataset splits). The Gulf of Mexico deployment was divided into coastal and deep ocean legs, which were phytoplankton- and zooplankton-dominated, respectively. The Norway dataset included imagery from Trondheimsfjord, Slettvik and Mausund, showcasing deep fjords and the Norwegian Sea. The Sargasso Sea deployment yielded data that were heavily zooplankton-dominated, particularly gelatinous clades.

The ISIIS-DPI uses an optical technique known as shadowgraphy, where objects passing through a collimated beam of light are captured with high definition as silhouettes in video (Figure 1). In this system, the light source is a blue (465–485 nm) LED. Thousands of 30 s videos were recorded at 22 frames per second, capturing an area of about 9 square inches with a pixel resolution of 2440 × 2048. Each pixel was approximately 35 μm tall. Knowing this, the taxa capable of being imaged ranged from 350 μm to 84.5 mm. In the final produced dataset, the taxa included ranged from 1.75 mm to 85 mm. Approximately 45 cubic inches or 0.74 L were imaged in any given frame. A segmentation and classification algorithm was developed using MATLAB, which was combined into a custom MATLAB application for streamlined processing (Supplementary Codes S1–S3). Due to the nature of shadowgraph imagery, water properties such as temperature and salinity gradients can appear as visible artifacts in the final video feed. Additionally, high phytoplankton or particle density can result in large regions of over-segmentation. With these challenges in mind, this current effort seeks to offer an adaptive and flexible segmentation methodology using ROI size, pixel intensity, and frequency thresholding. More specifically, training the four networks—i.e., Resnet, Xception, GoogleNet, and Darknet—using over 70,000 ROIs from three uniquely different marine ecosystems enabled enhanced performance when given out-of-distribution data not corresponding to images contained in the training dataset.

This project introduces a full algorithmic pipeline for the segmentation and classification of marine organisms in shadowgraph imagery. By using a select few base classes followed by modular niche networks, different water bodies with varying properties can be imaged and classified without the need for a large dataset upheaval. If instead, a single network was trained with a large array of classes, a new class addition would be impossible without retraining and reformatting the training dataset. The base classes chosen are typically common across all water bodies, with select regions requiring further class division. This way, the base class and network can remain robust, and further divisions can be implemented with no impact to the already developed network. Additionally, modular networks allow for a range of scientific goals to be accomplished. If a simple particle count is desired, the pipeline will filter some of the detrital or noise images and can quickly yield an organism count for each of the base classes. If instead, a researcher desired more in-depth statistics on a specific class, modular networks can be implemented in the pipeline, resulting in a longer computation time but a more descriptive output. As the base network is always used first, this also means that this further subdivision can be saved for a later date, for example, in the case of in-field research where time is a limited resource.

## 2. Materials and Methods

### 2.1. Data Acquisition

**Figure 2:**
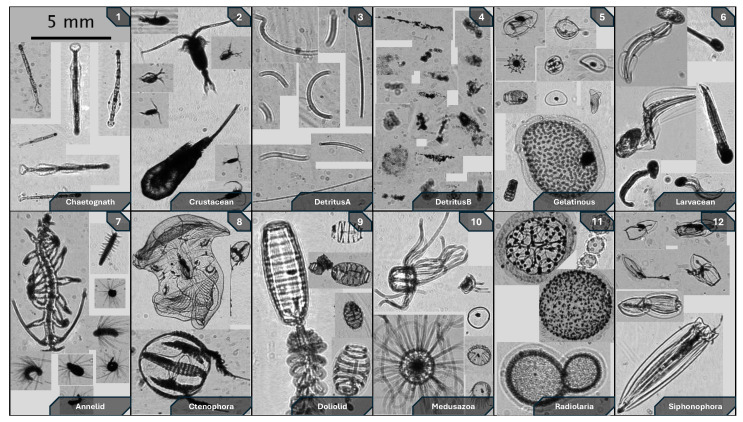
Sample images of planktonic groups present in dataset. (**1**) Chaetognath; (**2**) Crustacean; (**3**) DetritusA; (**4**) DetritusB; (**5**) Gelatinous; (**6**) Larvacean; (**7**) Annelid; (**8**) Ctenophora; (**9**) Doliolid; (**10**) Medusozoa; (**11**) Radiolaria; (**12**) Siphonophora. Subfigures (**1**–**6**) are members of the base dataset, while subfigures (**7**–**12**) are members of the secondary gelatinous dataset.

When deployed, the instrument frame would typically undergo a water column profile down to 100 m at descent rates of <1 m/s, then be brought back to sample the surface, i.e., 1–5 m depth, for the rest of the night. Shallower profiles down to 40–50 m were sometimes repeated throughout the sampling period to target specific layers of higher bioluminescence activity. The shadowgraph imager was recording at all times during these deployments. The on-board computer of the ISIIS-DPI, called SIDEKICK, was used for real-time view of the sampled volume to identify high biomass layers aboard the R/V. Videos were saved as 30 s clips at 22 frames per second, although recording rate often varied due to the on-board computer’s CPU being overtaxed. Figure 2 shows an assemblage of typical zooplankton sampled throughout all field deployments.

### 2.2. *Flat-Fielding*

**Figure 3:**
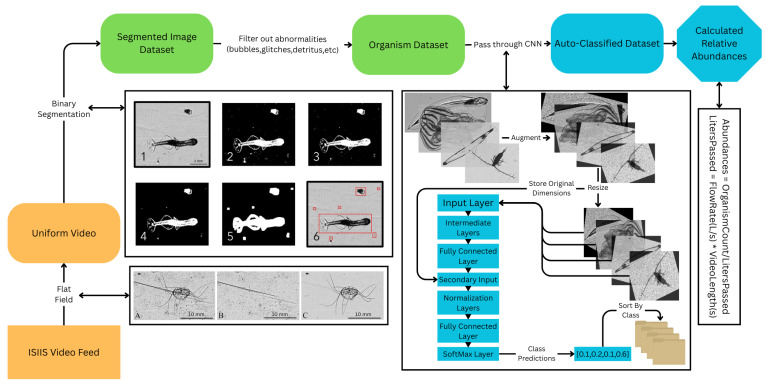
Full end-to-end algorithm. Orange blocks represent video data, green blocks represent bulk image datasets, and blue blocks represent neural network outputs. Flat-fielding is represented by the bottom left set of images, with steps being (**A**) base frame, (**B**) calibration frame and (**C**) uniform frame (Section 2.2). Segmentation is represented in the top left set of images, with steps being (**1**) raw frame, (**2**)binary thresholding (Section 2.3.1), (**3**) mask frequent pixels (Section 2.3.2), (**4**) remove small objects (Section 2.3.3), (**5**) region dilation (Section 2.3.4) and (**6**) region detection (Section 2.3.5). Classification is represented by the right set of images, with corresponding processes indicated on their respective arrows (Section 2.4).

Our approach is an end-to-end algorithm, meaning that segmentation and classification are both done by the algorithm, with human validation to determine accuracy and improvements. In summary, raw videos are first preprocessed to level the illumination and reduce background noise (Figure 3A–C). Following this, videos are binarized using binary thresholding, and the resulting output is further cleaned of noise and irrelevant regions (Figure 3, Sections 1–6). Videos are then ready to be sent through the full algorithm and have any objects present segmented and classified by the neural network.

The first preprocessing step is flat-fielding the raw PID video feed. This process applies an equation to each pixel to achieve a uniform luminosity across the frame. The formula can be expressed as follows:

(1) CorrectedPixVal=(RawPixVal−Offset)∗Gain

(2) Gain=AvgPixVal/CalibrationPixVal

Essentially, Corrected Pixel Value is the difference between the raw pixel and the offset value, multiplied by the gain. The offset pixel value is typically obtained from taking an image with the lens of the filter on; however, we took this value to be zero, as this is the darkest pixel value possible. While the true offset has some uncertainty attached, any effect it would have is negligible. The gain is the averaged value of that pixel across the whole video, divided by the pixel value in the calibration frame. The calibration frame is generated by sorting each pixel in the video and taking the average of the highest 80–90% of the pixel values. Bringing this all together, the simplified version of this can be written as:

(3) CorrectedPixVal=(RawPixVal∗AvgPixVal)/CalibrationPixVal

This process is represented in Figure 3A–C. After flat-fielding, the lighting may now be uniform, but there is potential for other artifacts to remain in the video. These artifacts, such as water temperature and salinity gradient, bubbles, or lens scratches, will be detected and segmented by the segmentation algorithm. To mitigate this, the video can be further processed, such as by applying filters or using image enhancement tools.

After the video background is satisfactorily uniform, the video can then be passed on to the next section of the algorithm, segmentation.

### 2.3. Segmentation

#### 2.3.1. Binarizing

The first step in segmentation is to convert the video to binary form using binary thresholding. This step is represented in Figure 3, with the raw image being shown in the first step and the raw binary image in the second step of segmentation. The binary thresholding process has maintained a position as the cornerstone of computer vision for decades, as it is extremely efficient. Each pixel value is compared to a defined threshold value, and if it is below said value, it is changed to one. Otherwise, if it is above or equal to that value, it is changed to zero. This threshold is typically chosen algorithmically, with our process utilizing the Otsu method [30]. This results in a binary image where only the darkest regions are turned on, which, in our case, will hopefully be zooplankton or other objects of interest. However, many of these ROIs will often be camera artifacts remaining from preprocessing. To mitigate these aberrations from proceeding further in the algorithm, the video is further processed.

#### 2.3.2. Mask Frequent Pixels

The binary video is run through another processing algorithm, where each pixel is given a frequency value determined by the amount of times that pixel is turned on in the binary video. These frequencies are compared against a user-defined maximum frequency, and turned off if the pixel exceeds this value. This mitigates many of the noise and artifact regions, while maintaining organisms that do not remain static in frame. For example, over 100 frames, if the top left corner pixel is 1 for 75 frames, it would have a frequency value of 75%, or 0.75. If the user inputs a maximum frequency of 0.6, this pixel would be deemed noise and set to 0 to avoid over-segmentation. The video then continues through the algorithm pipeline. This step is represented in Figure 3 in the third step of segmentation.

For our specific dataset, we have found that a value of 0.8 works best, with some mild tuning when required. This step is essential in the preprocessing algorithm. In the video feeds captured by the ISIIS-DPI, there will often be dark regions in the form of lens scratches or bubbles caught against the view port. These contaminating features can be more or less present depending on the type of deployment. If the imaging system is located near the air–sea interface and/or near pumps, bubbles will often be present in the field of view. These aberrations are typically static in place and, as a consequence, will have a high frequency value. These regions can then be effectively removed to avoid segmentation. There may be some fluctuation, resulting in lone pixels remaining; however, these are typically caught in the next step.

#### 2.3.3. Minimum Region Area

To further mitigate noise, any regions smaller than a user-defined area are removed from the video feed. This will remove any remaining lone pixels left in the previous step and remove any noise or detritus not large enough to be considered biologically relevant. This step is represented in Figure 3 in the fourth step of segmentation. This value should be given more credence over others, as a value too large may result in some of the smaller ROIs being lost. As with all of these values, it should be tuned before use for each dataset. Depending on the scope of the research effort, this parameter can be tweaked to effectively select the particle size distribution of interest, e.g., detritus or marine snow, phytoplankton, or macrozooplankton.

For our purposes, we used anywhere from 15 pixels to 50 pixels, depending on the noise and population present in the dataset. Some regions may have gaps, causing them to be detected as separate ROIs, which will lead to incomplete and patchy masks if removed. For this reason, the minimum area at this stage is typically left smaller, with more region thresholding occurring after the regions have been dilated.

#### 2.3.4. Region Dilation

Organisms in frame will often not be completely binarized, due to translucency or some other property, often observed with gelatinous plankton. The ideal threshold can be difficult to pinpoint precisely, as too high a threshold will result in excessive noise being picked up, and too low will result in large “gaps” in relevant ROI masks. To solve this issue, each pixel is dilated by a user-defined area, causing the chunks of object to rejoin and be detected as one ROI (Figure 3, step 5). Similarly, this value must be tuned by the user, as too much dilation will result in nearby ROIs being grouped into larger clusters by the algorithm.

This dilation is achieved through MATLAB’s “imdilate” and a binary disc structuring element. For our purposes, a disc with radius 5 is used to dilate the regions. MATLAB’s “imdilate” works by taking each pixel as a center point and placing the desired structuring element, in this case a disc, thus expanding the region by an amount equal to the disc’s radius.

#### 2.3.5. Region Detection

The final step of the segmentation process is the actual ROI detection (Figure 3, step 6). This is done via the “regionprops” function available in MATLAB. A scripted loop passes each frame through the function, where each region is then labeled by its index number and a bounding box is recorded, which is later used to segment the object from the full image.

The steps listed above can be found in Supplementary Code S1, along with other segmentation-focused MATLAB scripts.

### 2.4. Classification

#### 2.4.1. Dataset Preparation

Once the segmentation process had been completed on a satisfactory scale, segmented organisms were separated and organized into distinct classes for categorization. These classes went through several iterations for optimal classification accuracy and performance. The first iteration consisted of 6 classes: (1) Chaetognaths, (2) Copepods, (3) Gelatinous, (4) Larvaceans, (5) Cladocerans, and (6) Radiolarians. Copepods and cladocerans were then combined into one crustacean class, and radiolarians were absorbed into the gelatinous class. These categories produced the best first-level classification, even though they do not follow accurate taxonomic classification, most likely due to convergence, i.e., similarity, in morphological traits.

The final dataset produced for this project was version 6.1, with a significant version change occurring each time a class was added or removed, and a minor version change whenever new images were added with no change to the overall class structure. This dataset contained 6 classes, with 2 of these being detritus classes. These classes were (1) Chaetognaths, (2) Crustaceans, (3) Detritus A, (4) Detritus B, (5) Gelatinous, and (6) Larvaceans. Detritus classes included bubbles, marine snow and most phytoplankton chains, as our current focus was to look at zooplankton-associated bioluminescence. The two detritus categories were mostly separated by shape, i.e., round vs. chain aggregates.

**Figure 4:**
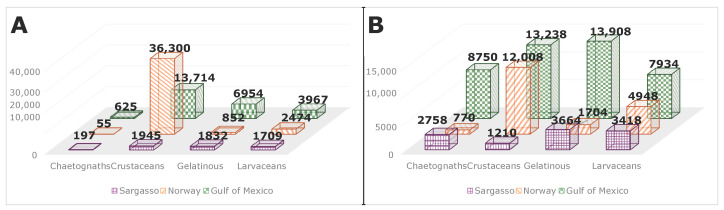
Non-augmented dataset distribution (**A**) and augmented dataset distribution (**B**) separated by category and field effort. Note different scaling on vertical axes.

**Figure 5:**
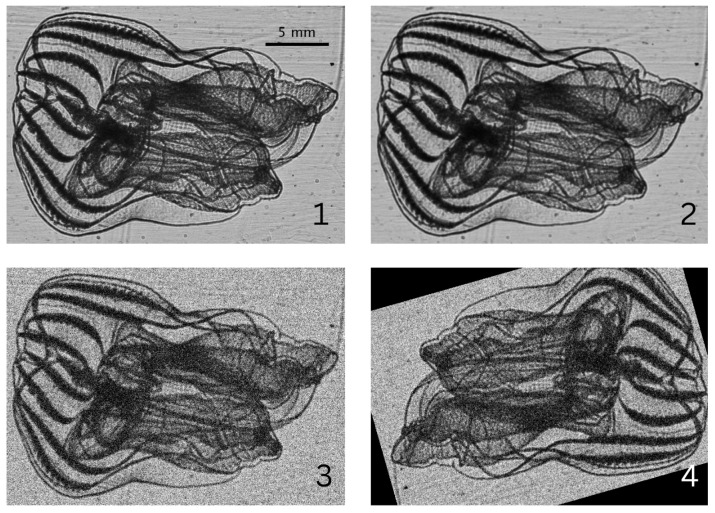
Augmentation process, with labels indicating: (**1**) base image, (**2**) Gaussian blur, (**3**) Gaussian noise, and (**4**) random scaling, rotation, and reflection.

This final dataset, pre-augmentation, consisted of 70,624 images, with class distributions shown in Figure 4A. As shown in the figure, this distribution was highly skewed toward the crustacean class, taking up more than 75% of the overall dataset. In an effort to make the dataset more uniform and improve network adaptability, the dataset was augmented following the steps shown in Figure 5. The base images were blurred using a slight Gaussian blur, followed by the addition of Gaussian noise. Finally, a set of scaling, rotation, and reflection was randomly selected and applied to the image. The number of augmentations for each class is shown in Table 1. Note that in the case of secondary niche datasets (discussed later in this section), the original raw images are used, i.e., augmentation is not applied twice. The number listed represents how many copies of the image, including the original, are present in the final augmented dataset. For example, looking at Figure 4A, the Sargasso Sea dataset has 1709 instances of Larvaceans. It has an augmentation factor of ×2, meaning that the augmented dataset (Figure 4B) contains the original image, plus an augmented copy.

Note the factor of ×0.5 for crustaceans. Due to their high volume, rather than augmenting and further increasing their strength in the dataset, half of the images were removed, with the larger images being prioritized. This produced a much more evenly split dataset, which was used to train the 4 network architectures. These results are discussed further in Section 3.1. The image augmentation pipeline can be viewed in its entirety in Supplementary Code S2.

The metrics we will be most interested in for network performance are precision and recall for each class, as well as overall dataset accuracy. To calculate these, 4 values are needed: the number of True Positive (TP)-, False Positive (FP)-, True Negative (TN)-, and False Negative (FN)-labeled images. For a multi-class problem, one way to calculate these values is to treat each class as a binary classification problem. For example, looking at the crustacean class vs. all other classes, TPs are the amount of correctly labeled crustaceans, while FPs are images incorrectly labeled crustacean. Similarly, TN refers to images correctly labeled as *not* crustacean, while FN refers to crustacean images that are incorrectly labeled as *not* crustacean.

The recall is defined as $\frac{TP}{TP+FN}$, and answers the question: “Out of all images belonging to class X, how many were predicted correctly?”. The precision is defined as $\frac{TP}{TP+FP}$, and answers the question: “Out of all images predicted as class X, how many were predicted correctly?”. A network’s overall accuracy is simply defined as the number of TPs for all classes, divided by the total dataset size. While the accuracy gives an idea of the overall network efficacy, precision and recall metrics provide greater insight into the network comprehension of each class.

#### 2.4.2. Neural Network Training

The final step of the algorithmic pipeline is classification of the segmented images. In this project, four network architectures were compared: (1) the ResNet-50 image classifier [31], (2) the Xception image classifier [32], (3) the GoogLeNet image classifier [33], and (4) the DarkNet image classifier [34], all available through MATLAB. These were chosen as they provided a well-rounded training group, as each network varies in structure, depth, and overall classification process. A more thorough analysis of each network’s architecture can be found in papers by He et al. [31], Chollet et al. [32], Szegedy et al. [33] and Redmon et al. [34], respectively.

Due to their varied nature, the networks all start with different input and output sizes, which needed to be corrected for our dataset. In this project, the number of classes was reduced from 1000 to 6, as well as adjusting the input size to match the grayscale 229 × 229 px images. While not typically conventional, 229 × 229 was chosen, as the network inputs all varied greatly and this value was located between the minima and maxima. Other input sizes were tested and compared against this value and yielded the same/slightly improved scores over other sizes. Note that by using the accompanying interface, the dataset size, as well as the input size of the network, can be adjusted by the user.

#### 2.4.3. Semi-Automatic Dataset Production

Following model training, a round of testing was done on unseen video data to determine the optimal network for automated classification. For the training dataset, Darknet performed best, but Xception proved to be the most consistent with out-of-distribution data. The trained model was saved to be loaded for further use.

This network was used iteratively on further video sets and locations, with a round of videos being segmented, classified, and validated by researchers, before being added to the growing image dataset. The dataset radially increased in size until the final dataset of 70,624 images was reached. After augmentation, the dataset included 91,354 images.

#### 2.4.4. Secondary Niched Networks

Following the classification of the main dataset into first-level categories, the gelatinous predictions were separated from the pipeline to be further classified into subclasses. These subclasses were based on conventional taxonomy rather than the top-level classes, which were based on the organism structural similarities. Currently, the only class where secondary classification was implemented is the Gelatinous class, due to its high intra-class variability. However, this can be extended to other classes with lower variability, given a sufficiently diverse and large dataset. These secondary categories were not implemented in the primary network, as this algorithm was designed to be versatile and modifiable depending on research goals and needs. In this sense, any main category can be divided into subcategories for secondary network training in the effort to answer specific questions, e.g., size spectra, taxonomic diversity, functional trait diversity.

**Figure 6:**
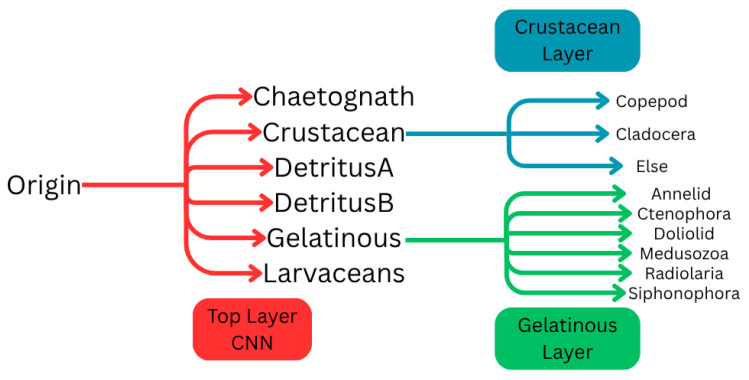
Base network with example sub-trees.

The secondary Gelatinous class was divided into 6 categories: (1) Annelid, (2) Ctenophora, (3) Doliolid, (4) Medusozoa, (5) Radiolaria, and (6) Siphonophora (Figure 6). The same data augmentation process as described in Figure 5 was applied to this secondary dataset. This secondary classification functionality is available in the graphical interface created for this project. In the advanced settings section of the classification panel, the user can add additional subclasses in addition to the original 6 classes. For each new subtree, a new CNN is required for predicting and separating the subclasses. These can be imported by the user, or alternatively, the application offers several pre-trained networks trained using the produced project dataset.

### 2.5. Graphical User Interface

**Figure 7:**
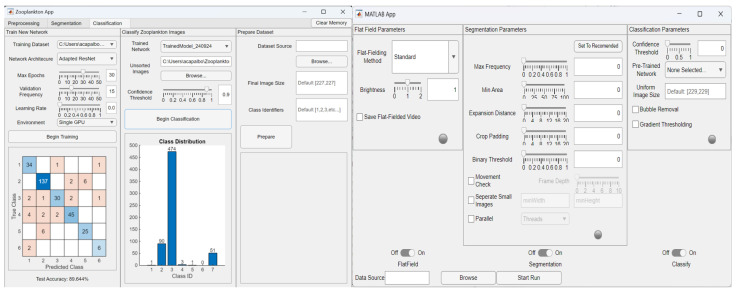
Example outputs in from the developed GUI, with standard mode being shown on the left, and batch mode on the right.

To better facilitate the algorithm process, a Graphical User Interface (GUI) was created using MATLAB’s App Designer (Figure 7). Through the GUI, users can import data as video files, with the option to apply all or select components of the algorithm.

Two iterations of this interface were developed, the first designed for passing a single video as input and the second acting as a batch job interface. Both interface application files can be found under Supplementary Code S3.

#### 2.5.1. Standard Mode

The standard interface mode allows users to parse through a single video frame by frame and find the optimal parameters for processing, segmentation, and classification. This is the first iteration of the interface, and features 3 tabs. The application opens on the first stage of the process, the preprocessing tab. There, a video can be loaded as an input and preprocessed using a number of tools, such as a Gaussian smoothing filter or a sharpening filter. This is where the raw video stream is flat-fielded and any processes to reduce noise are applied (Section 2.2). Once completed, the option to port the video to the next tab appears: segmentation.

The segmentation tab is responsible for locating and segmenting any ROIs passing through the frame. A search field allows a file to be loaded for segmentation if the user wishes to skip the preprocessing tab. Through the steps laid out in Section 2.3, the video is analyzed using a set of user-defined parameters, including maximum occurring pixel frequency, minimum object pixel area, and region dilation intensity. Once the ROIs have been saved to an external folder, along with a csv file containing their original bounding box positions, the user has the option to port the newly created dataset to the classification tab.

The classification tab allows the user to load pretrained neural networks bundled with the interface, which will then sort the segmented images into their predicted classes and write these to the user directory. Similarly to the previous tab, the user is able to port a dataset from the segmentation tab, or upload their own directory of images to be sorted. In addition to the sorting feature, the classification tab allows users to upload a labeled dataset of images and train a custom network based off the adapted network architectures used in this project. The user can import custom training parameters, as well as adjust the image input size and number of training classes.

#### 2.5.2. Batch Mode

Batch mode is the second iteration of the graphical interface, designed to take a set of parameters and apply it to an entire directory of videos. The user can select a single step of the process or any needed sequence, set the necessary parameters, and leave it to iterate over any number of videos. This is far more efficient, and allows for processing large amounts of data comparatively quickly. It offers the core features of standard mode, with only a few missing components, such as some of the optional filters available in standard mode.

Our plankton imager records 30 s files, yielding a video of ~600 frames. Per frame, we can expect anywhere from tens to hundreds of relevant ROIs passing through the field of view. Parsing through each round of video data on a singular basis would take hundreds of hours. The ability to move through these videos as “batches” has greatly reduced the overall computation and validation time needed for this scale of data.

### 2.6. Challenges

#### 2.6.1. Algorithm Imaging Limitations

This project introduces a unique set of challenges and limitations that must be taken into account. As with any imaging system, the ISIIS-DPI has an upper and lower bound for object size that can be imaged. Per the manufacturer, Bellamare: “You need at least 10 to 15 pixels to start representing an object adequately”. Therefore, the lower bound is roughly 350 μm, with the upper bound being the image dimensions, 2440 pixels, equivalent to 85,400 μm or 85.4 mm. Furthermore, organisms must be within the focus plane for the best resolution; the further they are imaged from this central plane, the more out of focus they appear. This unfortunately is an issue with any camera, as focus must be adjusted in any setting. As it is a shadowgraph imager, density or salinity differences are visible in the camera feed, resulting in excess detrital images whenever there is a high difference region.

The algorithmic pipeline, while robust, has its disadvantages as well. Binary segmentation is a foundational tool for computer vision, and is prevalent in many diverse fields. While powerful, if there is not a clear contrast between the foreground and background, segmentation will be sloppy and uneven. Flat-fielding the video feed corrects many instances of this, as a uniform background is necessary for ideal segmentation. This does not completely resolve the problem, as background regions in motion will become highlighted by the flat-fielding, rather than being “masked”. Attempts to mitigate this are discussed in the following section.

#### 2.6.2. Accounting for Camera Vibrations

During the flat-fielding process, we noted that there appeared to be an oscillatory movement to the background. After doing an analysis of the video feed, we found several layers moving independently of each other. The flat-fielding process assumes a static background, resulting in a “shading” effect that is detected as an object by the segmentation algorithm.

To correct this, the video was split up into 10 frame segments. With the oscillations occurring on a large enough time scale, the smaller range for the calibration frame was enough to mitigate the shading issues. While they can still appear in some frames, they are far more reduced than when they are subjected to the standard flat-fielding process.

An alternative method that was investigated was to move the calibration frame with the oscillations. While this did improve background shading slightly, due to the multiple different layers, the shake would not be consistent across the frame, resulting in inconsistent uniformity across each frame. In the end, this extra movement was deemed too computationally expensive to justify over the previous method.

#### 2.6.3. Out-of-Distribution Images

When training a neural network, it is only aware of the data it is shown. New, unseen data, termed out-of-distribution (OOD) data, can cause erratic predictions from the network. OOD images are typically camera aberrations, detritus, or unidentifiable objects. When these are fed into the network, the network is only capable of predicting one of its known classes. These incorrect predictions are varied, ranging from extreme confidence to near-random scoring. This proves detrimental to any automated system, as it is difficult to catch this type of error by the time it reaches the classification stage.

In this project, OOD images became quite a nuisance. It is impossible to know what will be encountered in the water column, and while the CNN can be trained on some common OOD images, this is only a temporary solution. The set of in-distribution images has simply been expanded, and a newly encountered species or odd detrital shapes will result in the same OOD image response.

There are many possible approaches to dealing with OOD data. A common method is adding detritus/noise classes. Training the network on possible aberrations is a valid method, which was partially implemented in this project. However, this only prepares the CNN for the chosen subset of OOD data. If an OOD object that has not been included in the detrital classes passes through, the network will be unprepared and likely misclassify it.

An additional layer of filtering was implemented as an attempt to mitigate these OOD images. Many of the camera aberrations had an odd and distinct spike in their gradient histograms, which could be identified and removed from the classification pipeline. This is not a perfect process, however, and some OOD images still had a chance to reach the network stage. This is still an active area of research, specific to a given imaging system and deployment operation, and will be further mitigated in future iterations.

#### 2.6.4. Dual-Input Adaptations

A consistent issue in the attempted automated classification was confusion for organisms with similar morphologies; this is a common issue with neural networks, but happened most consistently between the Chaetognath and Larvacean classes.

**Figure 8:**
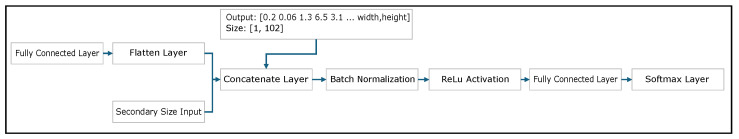
Size input adaptation for selected network architectures. The typical ending layers in these networks, a fully-connected to softmax layer, is replaced with a secondary input. This input takes an array of the original image sizes and takes them into consideration when classifying.

These classes do appear similar to one another; however, they have different sizes, with Larvaceans typically being much smaller than Chaetognaths. In order to be used as inputs, the dataset images must all be scaled to a uniform size, in our case, 229 × 229. In doing so, this scale difference is somewhat lost. This is a common issue with neural networks, and varied attempts at correcting this can be seen in other works, with Kyathanahally et al. [28] utilizing an additional feature set to compensate for the lack of size variation. In our case, a secondary input was added to the adapted network architectures (Figure 8). All of the architectures chosen for this project had a fully connected layer followed by a softmax layer, which generated the class predictions. To add a secondary input, a concatenation layer was used to join the original image size with the pre-classification weights. The new weights were then normalized and passed through an activation layer, where they were fed through the final fully connected and softmax layers.

A round of classification was done with two sets of networks; one set with the size adaptation, and another set with just the base architectures. The test data set was found to perform 2–4% better in the network with size adaptation, with ResNet specifically having an increase of nearly 5% in precision. Moreover, the precision between classes was much more consistent, with the confusion between larvaceans and chaetognaths being significantly reduced.

The MATLAB scripts used for this secondary input modification, as well as the base architectures themselves, can be found under Supplementary Code S2.

## 3. Results

### 3.1. Network Training Results

The final augmented dataset contained 91,354 images. A 70/15/15 train/validation/test split was used, meaning that 70% of the images were randomly selected for the training dataset, 15% for validation, and 15% for testing. The images were chosen uniformly, such that each sub-dataset provides an accurate representation of the total dataset. This split is commonly used in similar works [28], and was found to perform satisfactorily with the produced dataset.

As shown in Table 2, the adapted networks performed well with dataset v6.1, with all four final accuracies scoring in the range of 95% to 96%. In addition, precision and recall showed a well-rounded understanding of the dataset, with all values being over 92%. The Chaetognath and Crustacean classes seemed to perform best, both scoring above 95% for precision and recall.

The Gelatinous and Larvacean classes performed slightly lower, with the Larvacean class being below 95% for both recall and precision across all four networks. This is not too surprising, as of the four organism classes, these two had the most morphological variety, while the Chaetognath and Crustacean classes tended to have a more distinct and regular appearance. This is true for the detritus classes as well, with Detritus A performing slightly better across the four networks.

### 3.2. Niched Network Training Results

Following initial training on the version 6.1 base dataset, a new set of networks was trained and tested on the secondary niched network dataset for gelatinous organisms. The same four base architectures were used, these being Darknet, Google365, ResNet50, and Xception. The same image input and secondary input adaptations were performed on this set of networks as previously mentioned in Section 3.1. Additionally, the same 70/15/15 split as described in Section 3.1 was used on the secondary network dataset.

This iteration of networks had lower overall accuracy, recall and precision scores than the base dataset trainees; however, they all still scored fairly well, with accuracies ranging from 86.1% to 90.4%. Recall and precision scores were much more variable, spanning 63.2% to 96.9%. The two scores below 70% (Siphonophora recall 63.2%, Doliolida recall 63.5%) were both scored by the Darknet architecture. This is in contrast to the original dataset, where Darknet was one of the higher-scoring architectures. The base architecture set was extremely close overall, with all four networks scoring within 1% of each other. This set had more varied accuracies, with a range of 4%, but still performed well overall. The lowest-performing networks, ResNet and Xception, were now the top performing, with scores of 90.4% and 89.9%, respectively. These contrasts were likely due to decreased dataset size, as the base dataset consisted of 91,354 images, while the secondary dataset contained only 14,795 images. As the sample size grows, the networks will have more opportunity to learn the features unique to each class. Considering variable accuracies across networks, users could choose to use one CNN in particular for optimal classification, based on specific research interest.

### 3.3. Network Testing with Out-of-Distribution Data

Several videos from the Gulf of Mexico, unseen by the network, were run through the pipeline and sorted by the Xception network. Using a 70% confidence threshold, the images were sorted into their predicted classes. This yielded an observed instance count for each class. Looking at the predicted images in each class, a number of detrital and noise images went undetected by the preprocessing filter algorithm. This number varied depending on water properties and organism density, but remained consistent for a given video set. As discussed in Section 2.6, these unseen images are considered OOD, which the network is unprepared for. This issue was foreseen, hence the addition of detritus classes and pre-filtering. However, looking at the network recall, i.e., the ratio of correct class predictions to the total number of class members, the network scored very high. There were very few instances of misclassifications for the actual organism images, with the main errors being these OOD images. While abundances may be slightly overinflated (roughly 25%), their statistical relevance still holds. In the same water column, the same water properties will remain relatively consistent, meaning that the same proportions of OOD images should be consistent throughout a time series profile. This validates the use of these CNNs for ongoing field efforts and day-to-day biodiversity assessments without extensive and constant retraining.

**Figure 9:**
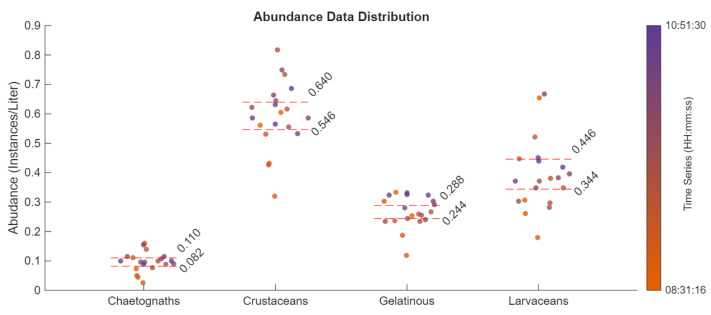

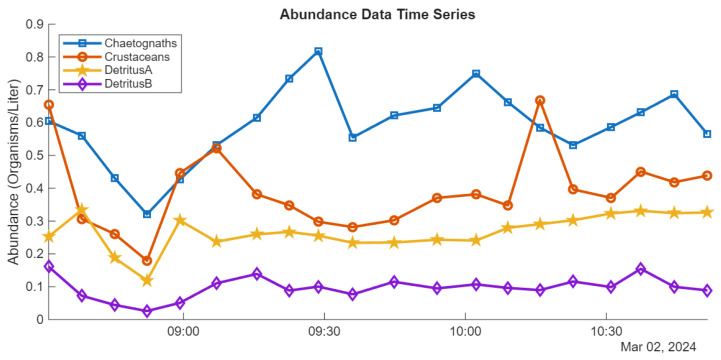
Abundance data extracted from ISIIS-DPI videos taken on 2 March 2024 (Florida Gulf Coast). Each data point corresponds to the number of ROIs identified normalized to volume imaged in a 30 s video, averaged over 10 data points. The color gradient indicates increase in time, with the first video starting at 08:28:04 AM UTC-6:00 and the last video starting roughly 2.5 h later at 10:59:52 AM UTC-6:00.

Knowing that these CNNs perform well with OOD data, they were subsequently used for autonomous estimates of planktonic diversity and densities. With a known flow rate of 18 L/s, i.e., the flow rate of our sampling package, a simple calculation yielded relative abundances for each class in units of organisms/L. This process was done for 101 videos taken by the ISIIS-DPI, giving 101 data points for each class. Outlier points outside of three standard deviations were removed, leaving 91 data points. Using MATLAB “bootci”, 95% confidence intervals were generated for each class. The corresponding outputs can be seen in Figure 9. The color gradient indicates an increase in time, with the first video starting at 08:28:04 AM UTC-6:00 and the last video starting roughly 2.5 h later at 10:59:52 AM UTC-6:00.

## 4. Discussion

Four neural network architectures were used to train CNNs and assess their viability in automated classification of marine planktonic clades. These architectures—ResNet, Xception, GoogleNet, and Darknet—are fairly common and provide a relative standard to test our unique dataset against. Additionally, their varied depth and structure offer the opportunity to test how different CNN structures interpret the training set.

The high average accuracy, e.g., 95–96%, in all four tested CNNs shows that these specific networks are adequate for classifying zooplankton, once isolated from frames using our segmentation algorithm. While all four networks averaged very similar accuracies (Table 2), GoogLe365 achieved the most consistent results and highest accuracy. The Larvacean category was, on average, the lowest-performing group. The wide range of organism sizes encountered in research cruises could be partly responsible for this occasional misidentification.

Additionally, contrast gradients had high variability for this category, since Larvaceans were often imaged while still in their mucus housing (Figure 2, Figure 3, Figure 4, Figure 5 and Figure 6). Indeed, when normalized to size, these two groups were morphologically similar, e.g., translucent body edges, large circular head, vermiform body. However, using image size, i.e., organism size, as a secondary input into the CNNs (Figure 8) greatly increased the accuracy of both the Chaetognaths and Larvaceans categories. In this sense, dual-input adaptations like image size can help CNNs differentiate classes with very similar morphological traits.

Training times varied greatly among networks. GoogLe365 had the lowest training time by far (70 min), whereas Darknet and Xception trained for 6 and 11 h, respectively. Additionally, GoogLe365 trained for the highest number of epochs until no improvement was reached. This correlates with network depth, where a network with more layers will take longer to iterate through one epoch compared to a shallower network. GoogLe365 has only 22 layers compared to Darknet’s 53. Consequently, fewer layers equates to fewer opportunities for CNNs to detect underlying patterns in the dataset, thus leading to more epoch iterations before improvement stagnates. While GoogLe365 performed best on the training dataset, when presented with unseen data, it had difficulty distinguishing OOD data with valid classifications. Other networks with deeper architectures, such as Xception or Darknet, appeared to perform better with OOD data. Shallower networks such as GoogLe365 may be best suited for tasks where only an estimate is required, but for tasks requiring low uncertainty, a deeper network may be advantageous. Additionally, other CNNs may be valuable when looking into specific categories, e.g., niched networks.

Comparable works have trained networks on datasets with many more base classes. Instead of training on a high number of categories simultaneously, we decided to use a hierarchical approach for more in-depth classification. Starting with six classes allowed for general counts to be obtained, with more specific species counts if desired. Indeed, as a proof of concept, a niched network was implemented in the gelatinous category, since this class contained the highest morphological diversity, e.g., medusoids, ctenophores, pelagic worms. The high precision and recall in secondary niched networks showed that autonomous classification following traditional Linnaean taxonomy (branching) is possible (Table 3). Given adequate dataset size, creating multiple hierarchical niched networks might allow for family- or even genus-level categorization. Of course, this implies enough morphological disparity between clades for CNNs to identify and train on. Additionally, using niched networks can reduce overall processing time, since specific clades can be targeted and classified based on scientific goals. In this sense, our approach aims to produce a versatile end-to-end algorithm that can be tailored to the user.

Future classification algorithms could implement an ensemble approach, where multiple networks “vote” on the most likely class. While this may increase time complexity, this approach has been shown to classify well in similar environments [28]. Multiple trained architectures in an “ensemble” would combine their softmax predictions into a new feature set, which is then used as an input for some form of final classifier. This could be another CNN, or another machine learning (ML) classifier such as a random forest [28]. Another potential method of incorporating multiple networks into the classification pipeline could involve placing binary classifiers at the final prediction layer [23]. The initial class predictions would be used as inputs, and each binary classifier would determine whether the image was a member of its target class or a misclassification. For example, the crustacean class binary classifier would take all of the initial crustacean images and make predictions if each image were a crustacean or a misclassification. This would be repeated for each class. Similarly to the previously mentioned approach, this implementation would increase time complexity, but would provide additional filtering of any OOD images that make it past the initial removal phase.

Additionally, future iterations including the nearest neighbor distances (NNDs) of ROIs within a single frame could yield valuable data and insight into zooplankton interactions in the water column, and if these patterns change over a temporal and spatial scale. While these questions have previously been studied [21,35], our algorithm would be capable of computing these NNDs automatically.

Combining these relative abundances with other data such as salinity, temperature, or acoustic data could allow for patterns to be learned by a new CNN. Using these properties as a feature set for other CNN types could potentially allow for abundance and species estimates to be made without the need for a complex planktonic imager. Moreover, the use of CNNs can facilitate multidisciplinary studies. In our specific case, the ISIIS-DPI was deployed upstream of a bioluminescence sensor. Once accurate networks had been established, we aimed to train the networks on recognizing key bioluminescence groups, e.g., appendicularians, ctenophores, large dinoflagellates, and krill. From there, abundance tables could be established (Figure 9 ) specifically on bioluminescent groups, and estimates of the water column’s latent potential of mechanically stimulated luminescence (Latent MSL) could be made at variable depth increments. These estimates have been made in the past, but require large sample sizes for adequate density measurements [36,37,38]. Coupled with CTD data, classification algorithms could uncover trends in planktonic bioluminescence and facilitate long-term monitoring of environmental and community changes [39].

Comparing with other similar works, others have employed similar strategies for their segmentation algorithms [28]; however, this is not always the case. Many projects instead opt for manual segmentation to build up a dataset, wherein a video or image feed is taken and bounding boxes are manually drawn on each ROI. Both routes have their advantages and disadvantages. Automated segmentation, while allowing for the segmentation of mass amounts of images, requires the researcher to validate outputs and make corrections when necessary. Manual segmentation avoids these issues of incorrect segmentation; however, the required segmentation by the researcher will negate the time saved in validation. For this project, we opted for automated segmentation, as our raw dataset was far too large to be processed by manual segmentation.

Approaches vary in terms of CNN implementation as well, with many opting for similar traditional CNN architectures [28,40]. These projects may have differing architectures, but still have comparable training parameters. Comparing results shows similar accuracies on their testing datasets. In contrast, many have begun implementation of semantic segmentation networks such as “U-net” [41]. These networks yield pixel-wise classifications, meaning they directly segment objects from frames while simultaneously classifying them. Once again, both routes have their advantages and disadvantages. Semantic segmentation, while a powerful tool, requires a dataset of full-image frames with each pixel labeled as their target class. For this project, a traditional bounding box segmentation algorithm followed by a CNN was implemented, as the ability to segment without necessarily classifying was desired. With this, a video could be segmented and analyzed for a qualitative estimate of the water column population. A semantic segmentation network, however, would only be able to view each frame and make its prediction values, and in the case of OOD data, it would likely be ignored and completely missed while in the field of view.

Reviewing the current literature, automated classification using out-of-distribution data is typically not discussed. This project is unique in the sense that this is an end-to-end algorithm, i.e., segmentation and classification are completed in one pass over the input video feed. Using this segmented output as a direct input to classifiers seems to be uncommon, with most projects opting for segmentation followed by labeling. This avoids the possibility of the network encountering OOD data, but leaves a large area of potential weakness when classifying new data. While automating classification and other tasks is the eventual goal of this technology, much work has yet to be done on applying these networks to real-world systems. OOD data remains an issue in many fields, with many projects dedicated solely to mitigating these issues in related classification tasks [42,43].

While autonomous segmentation can yield thousands of images in short amounts of time, the range of organisms is inherently limited by the instrument being deployed. Indeed, not unlike net mesh size dictating the size distribution and type of collected plankton [2,44], the imager’s optics, e.g., CCD chip size, lenses, and pinhole diameter, select toward a certain class size. In this sense, plankton abundances calculated using autonomous samplers will be biased on the basis of instrument limitations. For example, the ISIIS-DPI is optimized for ichthyoplankton and zooplankton [7,40,45], the Imaging FlowCytoBot is tailored for phytoplankton [46], the AUTOHOLO images phytoplankton and small zooplankton [6,9,18], and the ZOOCAM optimally samples zooplankton [4]. Like traditional sampling methods, an autonomous system cannot solely provide an accurate overview of the entire planktonic community, but when used in tandem, they can provide answers to problems typical sampling cannot [47].

The imaging system and proposed algorithm yield unique information regarding planktonic community composition, distributions, and functions and monitor local changes in organism density. By recording with the ISIIS-DPI at discrete depths and time intervals, one could use calculated abundances to get estimates on a population’s current trends. This could be done in many different forms, such as continuous monitoring stations or buoy deployments, but the core algorithm would remain the same [4]. Knowledge of species growth or decline could provide insight into blooming of phytoplankton populations, grazing patterns of larger zooplankton, community composition changes, as well as the overall water quality of the ecosystem. Autonomous segmentation and classification of zooplankton can be further advanced to study functional and behavioral traits [8]. Greer et al. [48] and Greer et al. [12] used multiple autonomous systems to successfully image spatial competition, and predation and parasitism events, most of which could not be studied with traditional sampling methods. Indeed, once ROIs are categorized into general taxonomic groups, niched networks could be tailored toward water column interactions like predation and grazing, sexual or asexual reproduction, and morphological variation within clades, e.g., zooid types in siphonophores, sexual dimorphism, gravid individuals, ontogenic stages. Thus, establishing an autonomous and modular segmentation and classification pipeline opens up several new avenues in zooplankton ecology, from the individual to the ecosystemic level.

## 5. Conclusions

In this project, a unique dataset was constructed from three distinct marine ecosystems: the Florida Gulf coast, Trondheimsfjord, Norway, and the Sargasso Sea. An adaptable segmentation algorithm was developed to be used for these and future locations. Using videos obtained with the ISIIS-DPI, over 70,000 ROIs were detected and segmented for future use. With these images, four CNNs of varying structure and depth were trained and tested for accuracy. Using datasets sampled from multiple diverse locations gives these networks the opportunity to learn large-scale features on a more global scale. It was found that these networks performed adequately on the training dataset. However, new images sampled directly from the ISIIS video feed proved challenging. The networks did well with objects that were present in the training dataset, but out-of-distribution data that made it past the initial filters negatively impacted the networks’ accuracies, and tended to overinflate automated abundance counts. This remains an issue, as out-of-distribution data is persistent in in situ environments. These OOD images can result in unpredictable outputs from the CNN, and are a major hindrance in any automated classification task. Future iterations incorporating multiple networks could mitigate these issues. As more locations are added to the networks’ repertoire, their capabilities in autonomous species analysis will only grow. In future works, incorporating depth data could lead to species abundance throughout the water column, giving researchers insight into species distribution, functional ecology, and potential thin layers of high species abundance [16].

## Figures and Tables

**Table 1:** Multiplicative factor used when augmenting base dataset (top) and secondary dataset (bottom). Secondary datasets use the original images, i.e., augmentation is not applied twice to any images.

Organism Class	Augmentations
Chaetognaths	×14
Crustaceans	×0.5
DetritusA	×14
DetritusB	×14
Gelatinous	×2
Larvaceans	×2
**Secondary Organism Class**	**Augmentations**
Annelida	×8
Ctenophora	×5
Doliolida	×8
Medusozoa	×2
Radiolaria	×3
Siphonophora	×7

**Table 2:** Training matrix showing precision and recall of the four networks for each category of the first level of classification. Yellow and green highlights show accuracies of 90–95% and >95% respectively. Total training times and number of epochs are also listed, with training terminating after 15 epochs or 50 iterations with no improvement.

Class		Network Architecture			
		Darknet	GoogLe365	ResNet	Xception
Chaetognath	Recall	95.01%	95.06%	95.66%	95.55%
	Prec	96.31%	96.05%	95.87%	95.29%
Crustaceans	Recall	99.12%	99.19%	99.17%	98.79%
	Prec	98.65%	98.60%	98.60%	99.04%
DetritusA	Recall	95.85%	96.07%	95.63%	95.85%
	Prec	94.34%	94.89%	95.42%	94.34%
DetritusB	Recall	94.85%	95.69%	95.02%	93.67%
	Prec	93.20%	92.49%	92.90%	94.54%
Gelatinous	Recall	94.40%	93.84%	94.26%	94.64%
	Prec	95.59%	95.70%	95.15%	94.70%
Larvacean	Recall	94.27%	94.68%	93.70%	94.27%
	Prec	94.31%	94.95%	94.55%	94.43%
**Accuracy**		96.01%	96.09%	95.97%	95.94%
**Time/Epochs**		6:04:16/11	1:10:21/15	1:38:28/11	11:03:29/11

**Table 3:** Training matrix showing precision and recall of the four networks for each category of the second level of gelatinous classification. Green highlights show >90%, yellow indicates 80–89%, and red indicates <80%. Total training times and number of epochs are also listed, with training terminating after 50 iterations with no improvement.

Class		Network Architecture			
		Darknet	Google365	Resnet	Xception
Annelid	Recall	88.66%	79.38%	93.81%	89.69%
	Prec	74.78%	96.25%	89.22%	88.78%
Ctenophora	Recall	75.00%	73.24%	80.99%	79.93%
	Prec	76.34%	88.14%	86.14%	82.85%
Doliolid	Recall	63.51%	87.84%	84.46%	88.51%
	Prec	86.24%	76.02%	88.03%	89.73%
Medusozoa	Recall	93.34%	93.20%	93.78%	92.19%
	Prec	84.42%	87.50%	88.16%	88.23%
Radiolaria	Recall	93.18%	96.15%	94.54%	94.67%
	Prec	94.11%	93.04%	95.97%	96.95%
Siphonophora	Recall	63.21%	68.91%	77.72%	77.72%
	Prec	79.22%	81.60%	83.80%	78.13%
**Accuracy**		86.12%	88.64%	90.40%	89.91%
**Time/Epochs**		1:53:27/23	0:19:45/40	0:45:12/36	5:09:38/33

## Data Availability

The original image dataset presented in this study are openly available upon request. Please reach out to the author at acapalbo@fau.edu.

## Supplementary Materials

The following supporting information can be downloaded at: https://github.com/acapalbo/ZooplanktonInterface; Code S1: MATLAB code for the segmentation algorithm: https://github.com/acapalbo/ZooplanktonInterface/tree/main/segmentationFunctions; Code S2: MATLAB code for classification algorithm: https://github.com/acapalbo/ZooplanktonInterface/tree/main/networkFunctions; Code S3: MATLAB code for the Graphical User Interface: https://github.com/acapalbo/ZooplanktonInterface/tree/main/interfaceFunctions. (All URLs were accessed on 7 March 2026).
